# 465. A Multifaceted and Multi-Institutional Analysis of the COVID19-Associated Mucormycosis Outbreak in the Delhi Area Indicates the Simultaneous Convergence of Multiple Risk Factors

**DOI:** 10.1093/ofid/ofac492.523

**Published:** 2022-12-15

**Authors:** Anuradha Chowdhary, Sebastian Wurster, Nitesh Gupta, Jason Mohabir, Shashidhar Tatavarthy, Vikas Mittal, Brijesh Sharma, Ying Jiang, Christina Cuomo, Dimiitrios P Kontoyiannis

**Affiliations:** Vallabhbhai Patel Chest Institute, University of Delhi, Delhi, Delhi, India; The University of Texas MD Anderson Cancer Center, Houston, Texas; Safdarjung Hospital, Vardhman Mahavir Medical College, New Delhi, Delhi, India; Genomic Center for Infectious Diseases at the Broad Institute of MIT and Harvard, Cambridge, Cambridge, Massachusetts; Artemis Hospital, Gurugram, Haryana, India; Max Super Speciality Hospital, Paschim Vihar, Delhi, India; Ram Manohar Lohia Hospital & PGIMER, New Delhi, Delhi, India; The University of Texas MD Anderson Cancer Center, Houston, Texas; Genomic Center for Infectious Diseases at the Broad Institute of MIT and Harvard, Cambridge, Cambridge, Massachusetts; The University of Texas MD Anderson Cancer Center, Houston, Texas

## Abstract

**Background:**

A major outbreak of COVID19-associated mucormycosis (CAM) in India in spring 2021 aggravated the death toll of COVID19. As the causes of that CAM outbreak remain unclear, we performed a multifaceted study of host, pathogen, environmental, and heath care-related factors in adult CAM patients (pts) in the metropolitan New Delhi area.

**Methods:**

We reviewed the records of all pts diagnosed with culture- or biopsy-proven CAM at 7 hospitals in the New Delhi area (April 1 – June 30, 2021). We used a multivariate logistic regression model to compare clinical characteristics of either all CAM cases (analysis 1, n = 50) or only pts with CAM after moderate or severe COVID19 (analysis 2, n = 31). As controls for both analyses, we used 69 COVID19-hospitalized contemporary pts. Selected hospital fomites were cultured for Mucorales. Additionally, we compared meteorological data and fungal spore concentrations in outdoor air before the CAM outbreak (January-March 2021) and during the outbreak (April-June 2021). Mucorales isolates from CAM pts were identified by MALDI-TOF-MS and ITS sequencing. A subset of 15 isolates underwent whole genome sequencing (WGS).

**Results:**

Risk factors for CAM in both analyses were newly diagnosed diabetes mellitus (odds ratio [OR] 8.26/5.67) and active cancer (OR 5.98/5.68) (**Figure 1**). Supplemental oxygen for COVID19 was associated with a lower CAM risk in both analyses (OR 0.13/0.17). Another significant CAM risk predictor identified only in analysis 1 was severe COVID19 (WHO score ≥ 6, OR 4.09), while remdesivir therapy (OR 0.40) and ICU admission for COVID19 were protective (OR 0.41) (**Figure 1**). No Mucorales were cultured from hospital fomites. The CAM incidence peak coincided with a significant uptick in environmental spore concentrations but was not linked to specific meteorological factors. *Rhizopus* was the predominant Mucorales genus (64%) identified by MALDI-TOF-MS and ITS sequencing; WGS found no clonal population of isolates but detected 2 cases of the rare pathogen *Lichtheimia ornata*.

**Figure 1:**
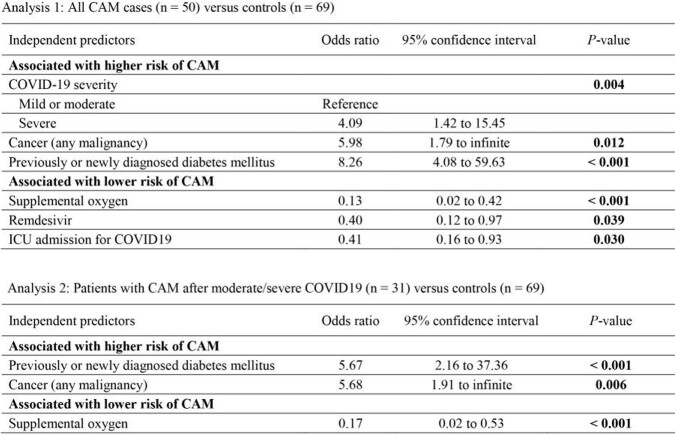

**Conclusion:**

An intersection of host, environmental, pathogen and healthcare-related factors might have contributed to the emergence of CAM. Surrogates of access to advanced treatment of COVID19 were associated with lower CAM risk.

**Disclosures:**

**Dimiitrios P. Kontoyiannis, MD, ScD, PhD (hon)**, AbbVie: Advisor/Consultant|Astellas Pharma: Advisor/Consultant|Astellas Pharma: Grant/Research Support|Astellas Pharma: Honoraria|Cidara Therapeutics: Advisor/Consultant|Gilead Sciences: Advisor/Consultant|Gilead Sciences: Grant/Research Support|Gilead Sciences: Honoraria|Merck: Advisor/Consultant.

